# Novel neutralizing human monoclonal antibodies against tetanus neurotoxin

**DOI:** 10.1038/s41598-021-91597-2

**Published:** 2021-06-09

**Authors:** Takeharu Minamitani, Karin Kiyose, Ryota Otsubo, Toshihiro Ito, Hiroki Akiba, Rika A. Furuta, Tsuyoshi Inoue, Kouhei Tsumoto, Masahiro Satake, Teruhito Yasui

**Affiliations:** 1 Laboratory of Infectious Diseases and Immunity, National Institutes of Biomedical Innovation, Health and Nutrition (NIBIOHN), 7-6-8 Saito-Asagi, Ibaraki City, Osaka 567-0085 Japan; 2 Laboratory of Immunobiologics Evaluation, Center for Vaccine and Adjuvant Research (CVAR), National Institutes of Biomedical Innovation, Health and Nutrition (NIBIOHN), 7-6-8 Saito-Asagi, Ibaraki City, Osaka 567-0085 Japan; 3 Laboratory of Pharmaceutical Integrated Omics, Department of Pharmaceutical Engineering, Facility of Engineering, Toyama Prefectural University, 5180 Kurokawa, Imizu, Toyama 939-0398 Japan; 4 Laboratory of Proteome Research, National Institutes of Biomedical Innovation, Health and Nutrition (NIBIOHN), 7-6-8 Saito-Asagi, Ibaraki City, Osaka 567-0085 Japan; 5 Laboratory of Advanced Biopharmaceuticals, National Institutes of Biomedical Innovation, Health and Nutrition (NIBIOHN), 7-6-8 Saito-Asagi, Ibaraki City, Osaka 567-0085 Japan; 6 Japanese Red Cross Central Blood Institute, 1-67 Tatsumi, Koto-ku, Tokyo, 135-8521 Japan; 7 Division of Advance Pharmaco-Science, Graduate School of Pharmaceutical Science, Osaka University, 1-6 Yamada-oka, Suita, Osaka 565-0871 Japan; 8 Center for Drug Discovery Research (CDDR), National Institutes of Biomedical Innovation, Health and Nutrition (NIBIOHN), 7-6-8 Saito-Asagi, Ibaraki City, Osaka 567-0085 Japan; 9 Medical Proteomics Laboratory, The Institute of Medical Science, The University of Tokyo, 4-6-1 Shirokanedai, Minato-ku, Tokyo, 108-8639 Japan; 10 Department of Bioengineering, School of Engineering, The University of Tokyo, 7-3-1 Hongo, Bunkyo-Ku, Tokyo, 113-8656 Japan

**Keywords:** Immunology, Microbiology

## Abstract

Tetanus is a fatal disease caused by tetanus neurotoxin (TeNT). TeNT is composed of a light chain (Lc) and a heavy chain, the latter of which is classified into two domains, N-terminus Hn and C-terminus Hc. Several TeNT-neutralizing antibodies have been reported, but it remains unclear which TeNT domains are involved in neutralization. To further understand the mechanism of these antibodies, we isolated TeNT-reactive human antibody clones from peripheral blood mononuclear cells. We then analyzed the reactivity of the isolated antibody clones to each protein domain and their inhibition of Hc-ganglioside GT1b binding, which is critical for TeNT toxicity. We also investigated the TeNT-neutralizing ability of isolated antibody clones and showed that an Hn-reactive clone protected strongly against TeNT toxicity in mice. Furthermore, combination treatment of Hn-reactive antibody clones with both Hc-reactive and TeNT mix (the mixture of Hc, Hn, and Lc proteins)–reactive antibody clones enhanced the neutralizing effect. These results indicated that antibody clones targeting Hn effectively neutralized TeNT. In addition, the use of a cocktail composed of Hc-, Hn-, and TeNT mix–reactive antibodies provided enhanced protection compared to the use of each antibody alone.

## Introduction

Tetanus is a common, fatal disease caused by infection with *Clostridium tetani*, a Gram-positive obligate anaerobe. The bacterium enters wounds in the form of spores that germinate to produce tetanus neurotoxin (TeNT). The toxin blocks the neuronal release of inhibitory neurotransmitters, including acetylcholine, resulting in characteristic symptoms such as spastic paralysis^1^. TeNT is synthesized as a single 150-kDa polypeptide and is subsequently cleaved to generate an active toxin. The active form of TeNT consists of a light chain (Lc, also called fragment A, 50 kDa) and a heavy chain (100 kDa) linked by a disulfide bond, and the heavy chain is composed of two domains, Hn (also called fragment B, 50 kDa) and Hc (also called fragment C, 50 kDa)^2^. Lc works as a protease to cleave molecules such as vesicle-associated membrane protein-2 (VAMP-2), which is a soluble neuronal N-ethylmaleimide-sensitive attachment receptor (SNARE) protein, resulting in the blockade of inhibitory neurotransmitter release^3,4^. Hn plays a functional role in the translocation of Lc into the cytosol of neuronal cells^5^. Hc is indispensable for TeNT binding to target cells through ganglioside receptors such as GT1b^6^.

Human tetanus immunoglobulin (TIG), which is derived from the plasma of vaccine-administrated donors, is an effective tetanus therapy^7^. However, due to the lack of source plasma and the risk of contamination with infectious materials, the TIG should be replaced with a recombinant antibody (rAb) product^7,8^. Compared to mouse antibodies, human antibodies have an advantage as a therapeutic agent because they do not require a difficult humanization process. Several TeNT-neutralizing human antibodies have been reported^7–11^, but it is still relatively unclear which targeted TeNT domains are required for these antibodies’ neutralization activity. In addition, it has been reported that combinations of different antibodies, such as TIG, lead to enhanced neutralizing activity^9^, but it is not well understood which antibodies in such mixtures contribute to neutralization. To address these questions we isolated TeNT-reactive human antibody clones and assessed their TeNT inhibitory activity.

## Results

### Isolation and characterization of TeNT-reactive lymphoblastoid cell lines (LCLs)

**Figure 1:**
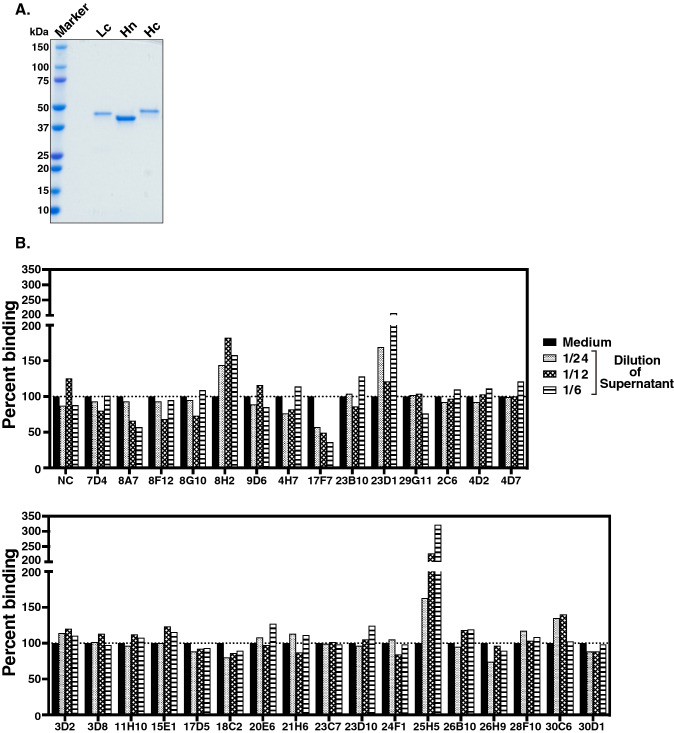
The 8A7 and 17F7 antibodies inhibit the binding of Hc to ganglioside GT1b. (**A**) The purified recombinant Lc, Hc, and Hn proteins were separated by 4–20% SDS-PAGE and stained with Coomassie Brilliant Blue (CBB). (**B**) The inhibition of Hc-GT1b by antibodies was analyzed by an ELISA-based Hc-GT1b binding assay. The supernatants of 31 Hc-reactive clones were serially diluted to 1/6, 1/12, and 1/24. Percent binding of Hc to GT1b is indicated.

To isolate monoclonal antibodies reactive to TeNT, we isolated peripheral blood mononuclear cells (PBMCs) from the peripheral blood of healthy volunteers, and infected these cells with Epstein-Barr virus (EBV), and established LCLs. Two weeks after EBV infection, we analyzed the reactivity of antibodies in LCL supernatants against the mixture of Lc, Hn, and Hc proteins (the ratio of Lc:Hn:Hc = 1:1:1, defined as the TeNT mix) (Fig. 1A) using an enzyme-linked immunosorbent assay (ELISA). We performed the screening experiments twice and obtained 18 and 35 TeNT mix–reactive LCL supernatants, respectively (Table 1). We further determined the reactivity of isolated LCL supernatants against Lc, Hn, or Hc protein by ELISA, and obtained a total of four LCL supernatants bound to the TeNT mix but not to Hc, Hn, or Lc proteins individually (TeNT mix–reactive supernatants) in addition to the supernatants bound to each domain (Table 1).

**Table 1:** Number of screened PBMCs and isolated LCLs.

Experiment ID	Screened PBMCs	Reactive LCLs				
		Hc	Hn	Lc	TeNT mix^a^	Total
1	4.7 × 10^7^	14	0	3	1	18
2	2.8 × 10^7^	17	3	12	3	35
Total	7.5 × 10^7^	31	3	15	4	53

^a^TeNT mix is composed of Hc, Hn, and Lc proteins. The number of TeNT mix–reactive supernatants indicates refers to clones that were not reactive to Hc, Hn, or Lc individually but were reactive to the mixture of these three antigens in ELISA.

### LCL supernatants, 8A7 and 17F7, inhibit the binding of Hc to ganglioside GT1b

Binding of the TeNT Hc domain to ganglioside GT1b plays a key role in TeNT toxicity, and inhibiting Hc-GT1b binding interfered with TeNT toxicity. To isolate the TeNT neutralizing antibodies, we performed an ELISA-based Hc-GT1b binding assay and evaluate the ability of Hc-reactive antibodies in LCL supernatants to block Hc binding to ganglioside GT1b. We analyzed 31 Hc-reactive LCL supernatants obtained in two rounds of screening experiments (Table 1), and found that two LCL supernatants, 8A7 and 17F7, inhibited Hc binding to GT1b in a dilution-dependent manner (Fig. 1B). In contrast, two LCL supernatants, 8H2 and 25H5, enhanced Hc-GT1b binding (Fig. 1B).

### Hn- or TeNT mix–reactive antibodies in LCL supernatants protect against TeNT toxicity in mice

**Figure 2:**
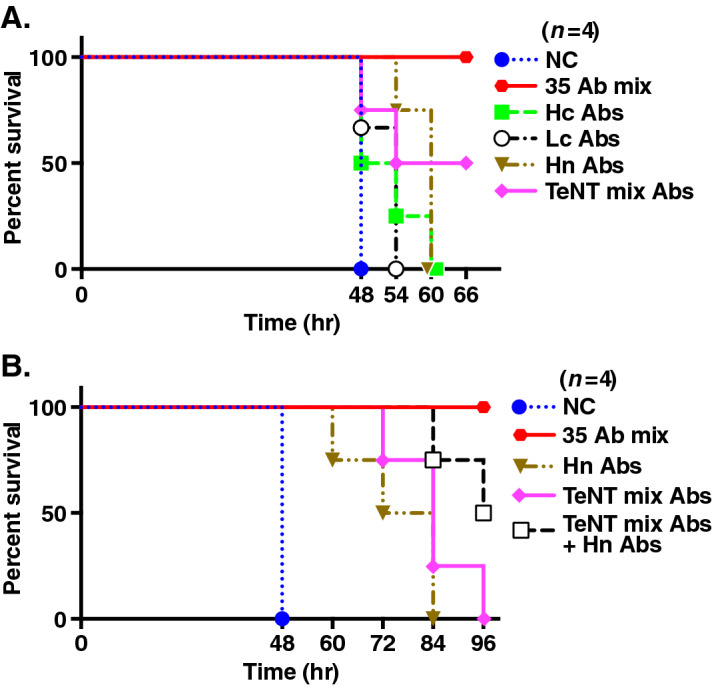
Hn- or TeNT mix–reactive antibodies in LCL supernatants neutralizes TeNT in mice. The protective effect of isolated antibody clones was analyzed by an in vivo TeNT-neutralization assay. We combined 0.5 ng human IgG-containing LCL supernatants from all 35 supernatants (35 Ab mix), 17 Hc-reactive supernatants (Hc Abs), 12 Lc-reactive supernatants (Lc Abs), 3 Hn-reactive supernatants (Hn Abs), or 3 TeNT mix–reactive supernatants (TeNT-mix Abs) prepared in a second screening experiment (Table 1). Then we mixed the supernatant mixture with 1 LD_50_ (25 pg) of TeNT and administrated it to ddY mice. Mouse survival rates after administration are shown (*n* = 4). *NC* negative control (medium). Statistical analysis was performed using Log-rank test.

To determine the target domain responsible for the neutralizing ability of antibodies, we performed an in vivo TeNT neutralization assay using LCL supernatants isolated in the second screening experiment, in which LCL supernatants reactive to all domains individually were obtained (Table 1). The mixture of all LCL supernatants (35Ab mix) completely neutralized TeNT (Fig. 2A). Mixtures of three Hn- or three TeNT mix–reactive supernatants (Hn Abs or TeNT mix Abs, respectively) significantly improved the survival of TeNT-treated mice compared to negative control (NC). (Log-rank test, *p* = 0.0082 or *p* = 0.040, respectively) (Fig. 2A). In addition, we examined the protective effect enhancement by the combination treatment with Hn- and TeNT mix–reactive supernatants and found the significantly increased protective activity compared to that of each Hn- or TeNT mix–reactive mixture alone (Log-rank test, *p* = 0.029 or *p* = 0.040, respectively) (Fig. 2B).

### Immunoglobulin gene usage analysis and characterization of recombinant antibodies

**Figure 3:**
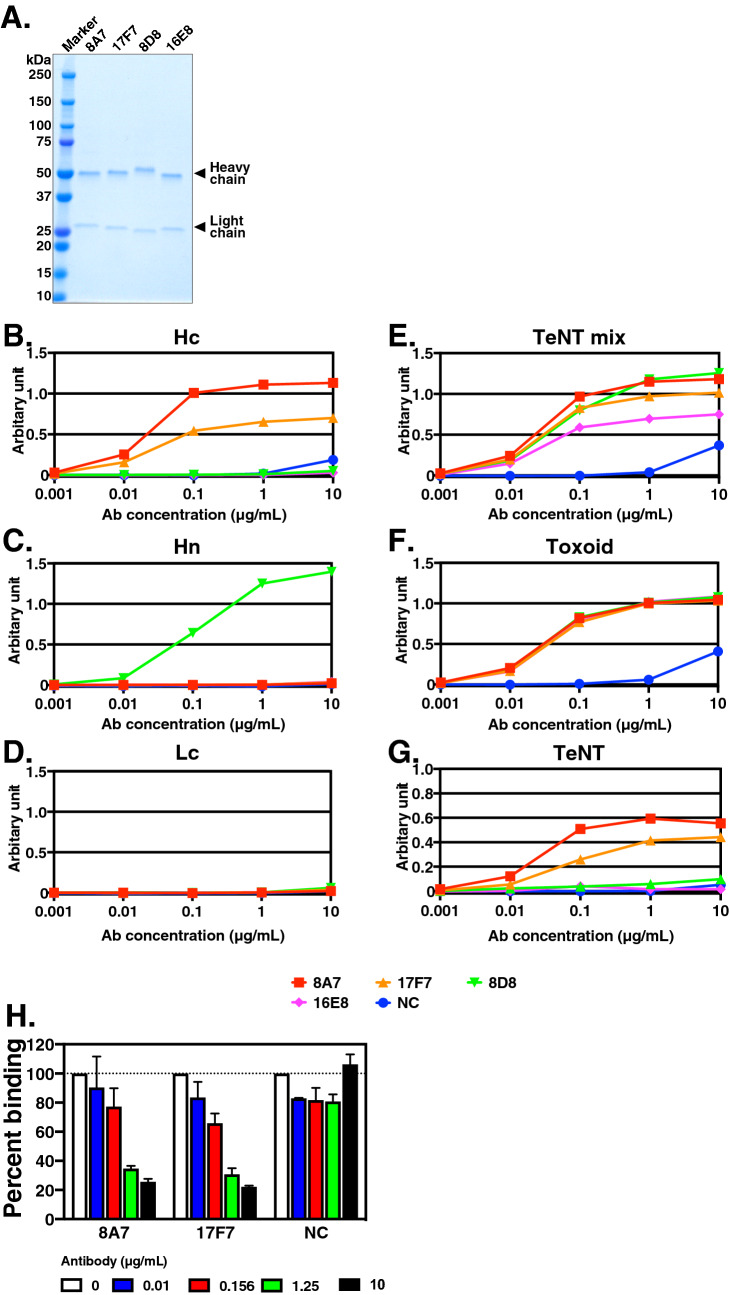
Reactivity analysis of recombinant antibodies by ELISA. The purified rAbs (8A7, 17F7, 8D8, and 16E8) were separated by 4–20% SDS-PAGE and stained with CBB (**A**). The reactivity of the rAbs against Hc (**B**), Hn (**C**), Lc (**D**), TeNT mix (**E**), tetanus toxoid (Toxoid; **F**) and native tetanus neurotoxin (TeNT; **G**) was analyzed by ELISA in triplicate. The concentration of antibodies was serially diluted from 10 to 0.001 µg/mL (from 64 to 0.0064 nM). Human IgG purified from human sera was used as a negative control (NC). (H) The inhibitory activity of Hc-GT1b by recombinant 8A7 and 17F7 was analyzed by an ELISA-based Hc-GT1b binding assay in triplicate. The concentrations of antibodies used for the assay were 0, 0.01, 0.156, 1.25, and 10 µg/mL (0, 0.064, 0.998, 8, and 64 nM). The percent binding of Hc to GT1b is indicated. Human IgG purified from human sera was used as a negative control (NC). The symbol or bar in each graph indicates the average. Error bars show the means ± s.d. Statistical analysis was performed using two-way ANOVA.

To characterize the isolated antibodies, we cloned the immunoglobulin heavy and light chain genes and analyzed their immunoglobulin gene usage and complementarity determining region (CDR) 3 sequence. As Hc-reactive antibodies, we cloned 8A7 and 17F7, which inhibited Hc-GT1b binding (Fig. 1B). As Hn-reactive and TeNT mix–reactive antibodies which had a neutralizing ability (Fig. 2A), we randomly selected 8D8 and 16E8, respectively and analyzed them. As a result, the V (D) J gene combinations and CDR3 amino acid sequences were differed between each clone, indicating that 8A7, 17F7, 8D8, and 16E8 had different origins (Table 2). We next generated and purified recombinant antibodies (rAbs) of 8A7, 17F7, 8D8, and 16E8 (Fig. 3A), and assessed their reactivity by ELISA (Fig. 3B–D). 8A7 and 17F7 reacted with Hc, but not Hn, and Lc, and 8D8 was reactive to Hn but not Hc, and Lc (Fig. 3B–D). All four antibodies bound to TeNT mix and toxoid in a dose-dependent manner (Fig. 3E,F). However, 8D8 and 16E8 showed very weak reactivity to TeNT (Fig. 3G). We also confirmed that purified recombinant 8A7 and 17F7 dose-dependently inhibited Hc-GT1b binding, and their inhibitory activity was significantly increased at the concentration of 1.25 and 10 µg/mL, compared to negative control antibody (NC) (two-way analysis of variance (ANOVA), *p* < 0.0001 for each) (Fig. 3H).

**Table 2:** Gene usage and CDR3 sequence of cloned immunoglobulins.

ID	VH	DH	JH	CDR3	Amino acid mutation (aa)
**IgH**					
8A7	IGHV3-33*03	IGHD6-19*01	IGHJ4*02	ARDKGYINGWYVPFFDY	15
17F7	IGHV3-33*01	IGHD6-13*01	IGHJ3*02	ARESGYASSWYFNGDAFDI	13
8D8	IGHV4-39*01	IGHD3-3*01	IGHJ5*02	ARLGVKKITLFGEVIPRSSWFAP	15
16E8	IGHV1-46*04	IGHD6-13*01	IGHJ4*02	ARDRRQQLVFDS	22

ID	Vk	Jk	CDR3	Amino acid mutation (aa)	
**IgL**					
8A7	IGKV3-15*01	IGKJ2*01	QQYDNWPPVT	4	
17F7	IGKV4-1*01	IGKJ4*01	QQYSSTPLT	5	
8D8	IGKV1-33*01	IGKJ5*01	QQYDTLSIT	3	
16E8	IGKV4-1*01	IGKJ3*01	QQYYSLSRGLT	9	

*CDR* complementarity determining region, *aa* amino acids.

### The mixture of several antibodies reactive to different domains exerts strong neutralization activity in mice

**Figure 4:**
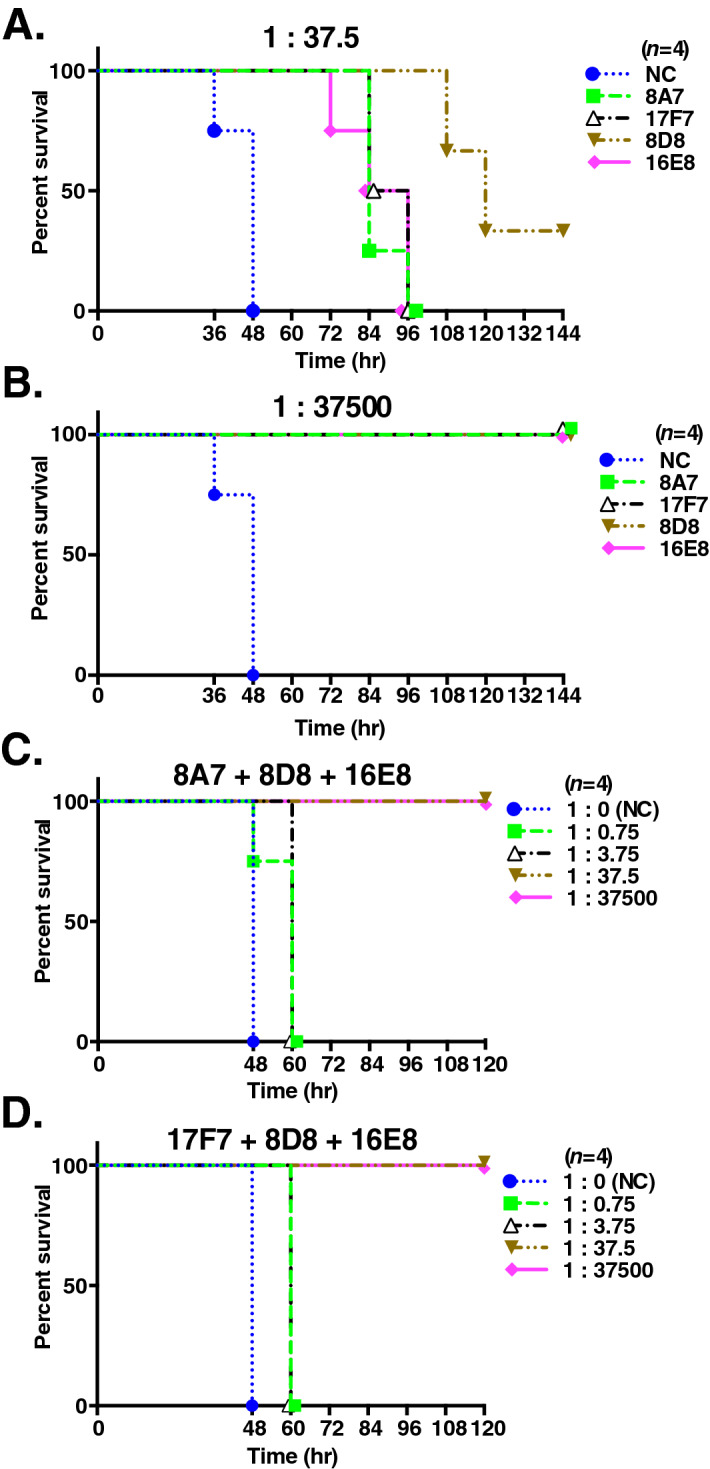
Mixing antibodies reactive to different domains completely protect the mice from TeNT. The TeNT-neutralization ability of purified recombinant 8A7, 17F7, 8D8, and 16E8 antibody clones were analyzed by an in vivo TeNT-neutralization assay. 1 LD_50_ (25 pg) of TeNT was used for the assay. (**A**,**B**) 3.75 ng (TeNT:rAb ratio 1:37.5) (**A**) or 3750 ng (TeNT:rAb ratio 1:37,500) (**B**) of each antibody was assessed. (**C**,**D**) The protective ability of the combination of 8A7, 8D8, and 16E8 antibodies (**C**) or 17F7, 8D8, and 16E8 antibodies (**D**) was analyzed. TeNT:rAb ratios of 1:0.75, 1:3.75, 1:37.5, and 1:37,500 were analyzed. Mouse survival rates following administration are shown (*n* = 4). *NC* negative control (PBS). Statistical analysis was performed using Log-rank test.

We assessed the TeNT-neutralization ability of purified rAbs in mice. For each antibody, 3.75 ng (TeNT:rAb ratio 1:37.5) significantly exerted protective ability against 1 LD_50_ TeNT compared to negative control (NC) in mice (Log-rank test, 8A7, *p* = 0.011; 17F7, *p* = 0.010; 8D8, *p* = 0.021; and 16E8, *p* = 0.010), and 8D8 showed the strongest protective activity in the four rAbs (Fig. 4A). In all cases, 3,750 ng of each antibody (TeNT:rAb ratio 1:37,500) completely protected the mice from 1 LD_50_ TeNT (Fig. 4B). We next prepared two antibody cocktails containing Hn-reactive 8D8, TeNT mix–reactive 16E8, and either Hc-reactive 8A7 or 17F7 to assess the neutralizing ability of combined treatment of antibodies. Both antibody cocktails targeting Hc + Hn + TeNT mix completely neutralized 1 LD_50_ TeNT toxicity in mice beginning at 1.25 ng each (3.75 ng in total, TeNT:rAb ratio 1:37.5) (Fig. 4C,D). The results in TeNT:rAb ratio 1:37.5 of Fig. 4A,C,D suggested that the neutralizing ability of each antibody was enhanced in combination with other antibodies.

### The neutralizing antibody 8D8 binds to the hydrophobic region of Hn

**Figure 5:**
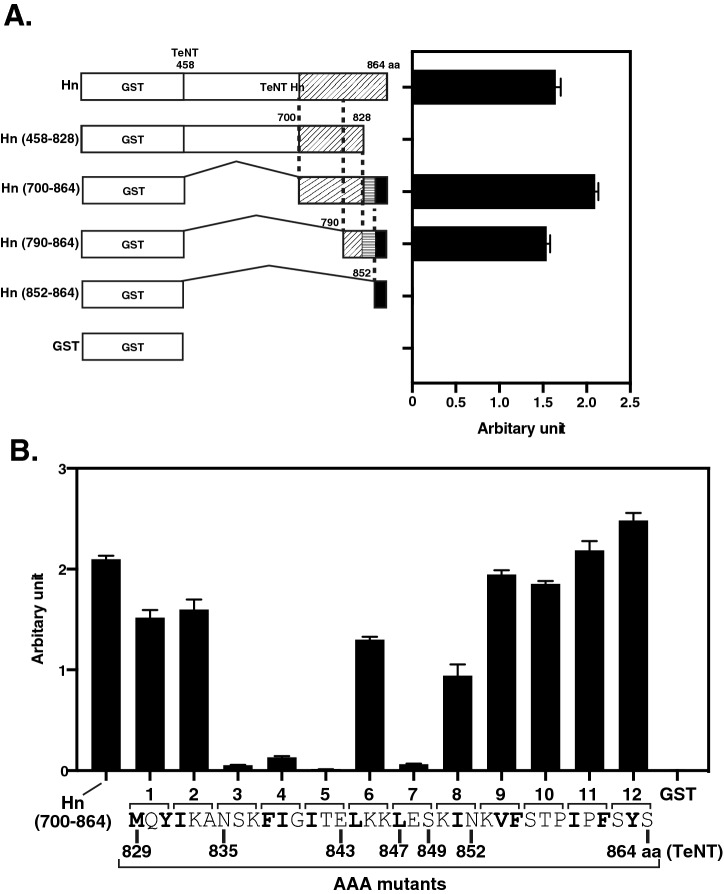
Epitope mapping of Hn-reactive 8D8. The reactivity of 8D8 to truncated Hn mutants (**A**) or Hn (700–864) mutants with triple alanine replacement (**B**) was analyzed by ELISA in quadruplicate. The amino acid sequence of Hn (829–864) is indicated and hydrophobic amino acids are shown in bold (B). The bar in each graph indicates the average. Error bars show the means ± s.d.

As shown in Fig. 4A, Hn-reactive antibody 8D8 remarkably neutralized TeNT toxicity in mice at relatively low dose. To deeply understand the molecular mechanism, we determined the binding site of 8D8 in Hn by ELISA. We first tested deletion mutants of Hn. Hn (458–828) showed no binding activity to 8D8, while this activity was retained by Hn (790–864) (Fig. 5A). We also prepared several additional mutants by replacing every three amino acids from 829 to 864 aa of the Hn (700–864) mutant with alanine and then examined the antibody’s binding ability (Fig. 5B). Hn (700–864) AAA.3, 4, 5, and 7 mutants lost binding to 8D8 (Fig. 5B), indicating that the regions from 835 to 843 aa and from 847 to 849 aa of Hn are important for 8D8 to recognition of Hn.

**Figure 6:**
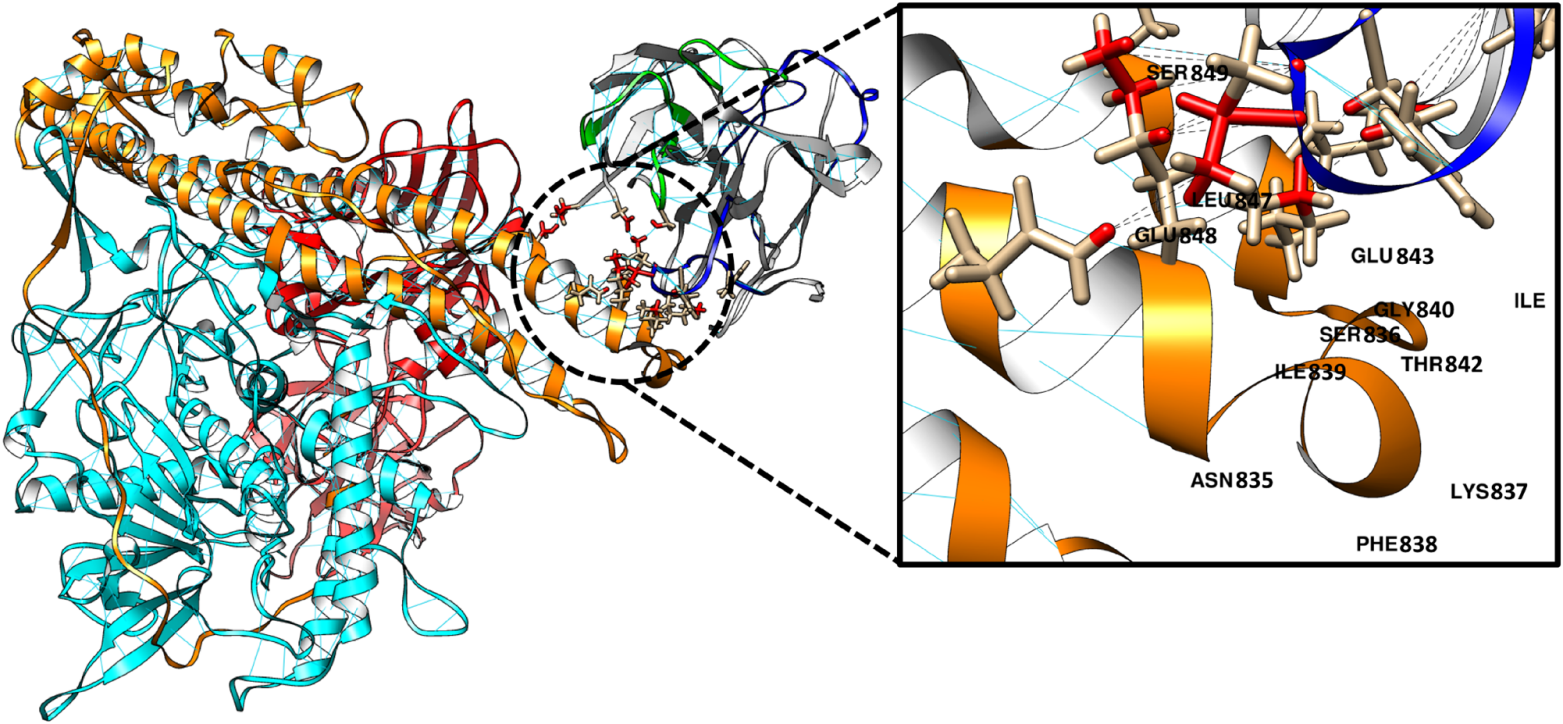
Docking model of Hn-reactive 8D8 with full-length TeNT. The docking model of Hn-reactive 8D8 with full-length TeNT (PDB ID: 5N0B) was obtained by using SnugDock software contained in Rosetta software suite (version 2020.37) (http://www.rosettacommons.org/). The 3-D structure of the variable domain of 8D8 was predicted by using RosettaAntibody software. A whole structure view and a close-up view of 8D8 and TeNT are shown by ribbon models, and experimentally determined binding sites (NSKFIGITE and LES) are shown by stick models with 3-letter amino acid codes and numbers. CDR loops in V_L_ and V_H_ of 8D8 are indicated with green and blue, respectively. Lc, Hn, and Hc domains in TeNT are indicated with cyan, orange, and red, respectively. Hydrogen bonds are indicated with cyan lines. Antibody-antigen interaction sites within a 5 Å distance are indicated with black dashes.

For more comprehensive assessment for these observations, we applied a computational approach to predict the variable domain structure of 8D8 and its recognition domain in TeNT. The computational simulation of antibody-antigen docking revealed that the mode of 8D8-Hn binding was similar to that from the analysis of the ELISA-based binding activity (Figs. 5 and 6).

## Discussion

TIG is widely used to treat tetanus and is known to be effective. This indicates that vaccinated individuals have TeNT-neutralizing antibodies in their peripheral blood. We hypothesized that we would be able to isolate neutralizing antibody clones from the PBMCs of such individuals. In this study, we modified a standard EBV infection method and established LCLs to isolate antibody clones. Then we cloned immunoglobulin heavy and light chain genes from the RNA of the LCLs. Using this strategy, we successfully isolated 53 TeNT mix–reactive LCL supernatants and identified four TeNT-neutralizing human antibodies, namely 8A7, 17F7, 8D8, and 16E8, from the PBMCs of a healthy individual. The healthy volunteer belongs to a presumed population that has been vaccinated against tetanus, suggesting that the isolated antibodies are derived from affinity-matured B cells. All four isolated antibodies had TeNT neutralizing activity, and completely protected mice from TeNT-induced death at high dose. In addition, the results of experiments treated with each antibody or combination of the four antibodies suggested that treatment with mixture of antibodies reactive to different domains of TeNT enhanced the neutralizing ability.

In the present study, we isolated a total of three Hn-reactive LCL supernatants from 53 TeNT mix–reactive supernatants. Compared to Hc- or Lc-reactive supernatants, fewer Hn-reactive supernatants were isolated. It has been reported that the Hn domain is hidden by the other domains due to the three-dimensional structure of TeNT^12^. Interestingly, Hn-reactive recombinant 8D8 did not bind to TeNT in our study. Botulinum neurotoxin (BoNT) is another highly potent toxin and is composed of three domains responsible for receptor binding (Hc), toxin translocation into cells (Hn), and proteolytic cleavage of host cell proteins (Lc), respectively^13^. It has been reported that most BoNT-neutralizing antibodies recognize Hc^14,15^. In the study, a small number of Lc-reactive protective antibodies were also isolated, but few antibodies bound to Hn domain^14,15^. In the case of diphtheria toxin (DT) which is consist of three domains, catalytic (C) domain, transmembrane (T) domain, and receptor binding (R) domain, Wenzel et al*.* recently reported that all three domains are targets for neutralizing antibodies and the T-domain-reactive antibodies showed the lowest neutralization potency^16^. Nevertheless, in this study we found that 8D8 bound to Hn via both Hn (835–843), in which hydrophobic amino acids form a cluster, and Hn (847–849), leading to TeNT-neutralizing activity in mice. Considering the reported three-dimensional (3-D) structure of TeNT, the region from 835 to 849 aa of TeNT could be relatively antigenic as it is externally exposed^12^. These points suggest that this region of Hn is a good target for neutralizing antibodies despite the low immunogenicity of Hn in humans. The computational simulation results of antibody-antigen docking in this study supports these agreements, because the simulation results gave a closer match to the experimental results of ELISA-based binding assays.

On the other hand, we isolated a total of four TeNT mix–reactive LCL supernatants, including 16E8, that did not bind with each Hn, Hc, or Lc in ELISA, suggesting that these antibodies recognize TeNT in conformation-dependent manner or the epitopes existing across the three domains. In fact, recombinant 16E8 bound to the TeNT mix and to toxoid, but not TeNT, native toxin, suggesting that the conformation of TeNT differs from that of TeNT mix and toxoid, and TeNT mix could form a structure similar to toxoid in our ELISA conditions. However, recombinant 16E8 antibody protected mice from TeNT toxicity as strongly as recombinant 8D8. Volk et al. also reported a protective mouse monoclonal antibody derived from tetanus toxoid-immunized mice that reacted with toxoid but not TeNT in ELISA^17^. Moreover, it has been reported that the structure of TeNT is changed by pH^12^, and thus it may be different in an ELISA assay than in vivo. These findings indicate that to select neutralizing antibody candidates in vitro, it is important to use ELISA to analyze the reactivity of antibody clones to the mixtures of each domain protein and to toxoid, as well as to each domain protein individually.

We also isolated a total of 31 Hc-reactive LCL supernatants, and two of these, 8A7 and 17F7, were the only clones that inhibited the binding of Hc to ganglioside GT1b in vitro. On the other hand, we observed enhancement of Hc-GT1b binding both by 8H2 and 25H5. Fitzsimmons et al*.* also reported an antibody that promoted Hc binding to GT1b^18^. This binding enhancement might be due to a conformational change in Hc. Recombinant 8A7 and 17F7 reacted with toxiod and TeNT in ELISA and had TeNT-neutralizing activity in vivo. Because the interaction of the TeNT Hc domain with GT1b is a critical to TeNT toxicity, blocking this interaction is thought to be very important for neutralization. To isolate TeNT-neutralizing antibody clones, therefore, it would be useful to examine whether antibody clones inhibit the binding of Hc to GT1b in vitro. In addition, Felix, L. Y. et al*.* has been reported the synaptic vesicle protein 2 (SV2) as a neuron receptor of TeNT^19^. It would be worthwhile to analyze the antibodies that inhibit the binding of TeNT to this receptor.

The combined supernatants of 35 TeNT mix–reactive LCL from our second screening experiment containing 0.5 ng of each antibody completely protected mice from 1 LD_50_ TeNT. Of the various mixtures of domain-reactive LCL supernatants, the Hn or TeNT mix–reactive mixture conferred the protection. The addition of Hn-reactive supernatants to the TeNT mix–reactive supernatants enhanced the latter’s protective ability, but unlike the 35Ab mix, it did not provide complete protection. These results suggest that in addition to the Hn- and TeNT mix–reactive antibody mixtures, other domain-reactive antibody clones are required for complete protection in our assay. Aliprandini et al*.* recently reported that the combination of Lc-, Hn- or full-length TeNT-reactive human antibodies conferred complete protection against TeNT in mice^20^. In fact, mixture of Hc-reactive 8A7 or 17F7, Hn-reactive 8D8, and TeNT mix–reactive 16E8 completely neutralized 1 LD_50_ of TeNT in our mouse experiments.

Finally, we isolated TeNT-neutralizing antibody clones from the PBMCs of a healthy individual using a modified EBV infection method. An in vivo TeNT neutralization assay revealed that the Hn-reactive and TeNT mix–reactive antibody clones had a protective ability and the combination of rAbs reactive to Hn, Hc, and TeNT mix enhanced TeNT-neutralizing activity. Collectively, although it is required for further experiments, such as treatment of antibodies after TeNT administration, and comparison of their neutralizing ability with TIG and previously reported human antibodies, the cocktail of the recombinant neutralizing antibodies identified in this study could be of help to the therapy for TeNT-induced infectious diseases instead of TIG.

## Methods

### Cell culture and reagents

B95-8-ZHT cells which are derived from B95-8 marmoset cells and stably express EBV BZLF1 in a 4-hydroxytamoxifen-dependent manner^21^, were cultured in RPMI1640 (Nacalai Tesque, Kyoto, Japan) supplemented with 10% fetal bovine serum (FBS; Merck, Darmstadt, Germany), streptomycin/penicillin, and 2-mercaptoethanol (Nacalai Tesque). LCLs were cultured in RPMI1640 (Nacalai Tesque) supplemented with 20% FBS (Merck), streptomycin/penicillin, 2-mercaptoethanol (Nacalai Tesque), K3 (Ajinomoto Bio-Pharma Services, Osaka, Japan), Ciclosporin (Novartis Pharma, Basel, Switzerland), IL-6, and BAFF (R & D systems, Minneapolis, MN, USA) (LCL medium).

### EBV preparation

To prepare EBV (B95-8 strain), B95-8-ZHT cells were treated with 400 nM 4-hydroxy-tamoxifen (Merck) for 5 days and then collected the cultured conditioned medium as EBV stock.

### Human blood samples

The blood donor was a healthy volunteer belonging to a presumed population that has been vaccinated against tetanus. Ethical approval for the study was obtained from the Institutional Review Board (IRB) of National Institutes of Biomedical Innovation, Health and Nutrition (NIBIOHN) (approval number 198), and informed consent was obtained from all participants. The study was performed in accordance with the guidelines of the Declaration of Helsinki.

### Mice

Four-week-old ddY mice were purchased from SLC Japan, Inc (Izu, Japan). The mice were used 1 week after purchase. All mice were maintained in a specific pathogen–free animal facility in accordance with the Osaka University guidelines for animal experimentation.

### EBV infection and LCL establishment

PBMCs were separated from whole blood by gradient centrifugation method using Lymphoprep reagent (Abbott Diagnostics Technologies AS, Oslo, Norway). Then IgM^+^ B cells were depleted from the PBMCs using Magnetic Cell Sorting-based anti-human IgM Microbeads according to the manufacturer’s protocol (Miltenyi Biotec, Bergisch Gladbach, Germany). The IgM^+^ B cell-depleted PBMCs were suspended in EBV stock (10^7^ cells/mL) and rotated at 37 °C for 1 h. The cells were then suspended in LCL medium (5 × 10^4^ cells/mL) and seeded at 200 µL/well in round-bottomed 96-well plates (Thermo Fisher Scientific, Waltham, MA, USA). After 2-week culture, the supernatant and the cells were collected, and ELISA and RNA isolation were performed.

### Purification of recombinant TeNT subunit and domain proteins

Plasmid DNAs encoding TeNT Lc (1–424 aa), Hn (458–864 aa, C467S), and Hc (865–1315 aa) were purchased from Eurofins Genomics (Luxembourg), and the protein-encoding regions were each inserted into a pCold GST expression vector (Takara, Shiga, Japan) by homologous recombination using the In-Fusion system (Takara) (pCold GST Lc, pCold GST Hn and pCold GST Hc, respectively). BL21 Star (DE3) (Merck) was transformed with each expression plasmid and cultured in Luria Broth medium supplemented with 200 μg/mL ampicillin at 37 °C. Expression was induced by the addition of isopropyl β-D-1 thiogalactopyranoside to a final concentration of 0.5 mM at an OD600 of ~ 0.5 with cooling to 15 °C. After expression for 24 h, cells were harvested at 4 °C by centrifugation for 20 min at 12,000×*g* and were stored at − 80 °C.

Frozen cell pellets were resuspended in a 1:5 (w/v) ratio of lysis buffer (50 mM Tris–HCl, pH 8.0, 200 mM NaCl, EDTA-free complete protease inhibitor cocktail tablets [Roche, Basel, Switzerland]), and lysed using an EmulsiFlex-C3 homogenizer (Avestin Inc., Ottawa, Canada), and centrifuged for 30 min at 20,000×*g* and 4 °C. Then the supernatant was filtered using a 0.45-μm syringe filter (Sartorius, Gottingen, Germany) and applied onto two 5-mL His Trap TALON columns (Cytiva, Marlborough, MA, USA). The proteins were eluted by 20 mL of an elution buffer (20 mM Tris–HCl pH 8.0, 200 mM imidazole, 200 mM NaCl). The hexa-histidine and glutathione S-transferase (GST) tags were removed by adding HRV3C protease directly to the eluent, which was then dialyzed overnight against phosphate-buffered saline (PBS). The protease and cleaved product were removed by a GSTrap HP column (Cytiva) in PBS containing 5 mM 1,4-dithiothreitol (DTT). The unbound fraction was dialyzed twice against 10 mM Tris–HCl pH 8.0 for 3 h, and purified by a HiLoad 16/60 Superdex 75 prep-grade column (Cytiva) equilibrated with size-exclusion buffer (10 mM HEPES pH 7.4, 100 mM NaCl). The purity of the purified proteins was confirmed by sodium dodecyl-sulfate polyacrylamide gel electrophoresis (SDS-PAGE).

### Expression of recombinant TeNT Hn mutant proteins

Expression plasmid vectors for TeNT Hn mutants were constructed using a KOD-Plus-Mutagenesis Kit according to the manufacturer’s protocol (Toyobo, Osaka, Japan). The primers used for PCR are shown in Table 3. The method for expressing proteins in *E. coli*. is described above. The proteins were extracted using 1% sodium deoxycholate, 5 mM EDTA pH8.0, and PBS as lysates. The cell lysates were used for capture, and ELISA was performed to determine the 8D8 antibody binding region for the Hn protein.

**Table 3:** Primers used to construct Hn mutant expression vectors.

Clone name	Primer 1	Primer 2
Hn (458–828)	TAACTCGAGGGATCCGAATTC	CAGGATGTTTTTTGACTGGG
Hn (700–864)	CATATGTCCCGGGCCCTGGAACAG	ATTATCAAAACTATCGACAACTTC
Hn (790–864)	CATATGTCCCGGGCCCTGGAACAG	ATGATTAACATCAACATCTTCATG
Hn (852–864)	CATATGTCCCGGGCCCTGGAACAG	AACAAAGTATTCAGTACCCCC
AAA.1	GCAGCAGCAATTAAAGCGAACTCCAAATTC	CAGGATGTTTTTTGACTGGG
AAA.2	GCAGCAGCAAACTCCAAATTCATTGGGATC	GTACTGCATCAGGATGTTTTTTG
AAA.3	GCAGCAGCATTCATTGGGATCACCGAACTC	CGCTTTAATGTACTGCATCAGG
AAA.4	GCGGCCGCGATCACCGAACTCAAGAAACTG	TTTGGAGTTCGCTTTAATGTAC
AAA.5	GCGGCCGCGCTCAAGAAACTGGAAAGCAAG	CCCAATGAATTTGGAGTTCGC
AAA.6	GCGGCCGCGCTGGAAAGCAAGATCAACAAAG	TTCGGTGATCCCAATGAATTTGG
AAA.7	GCGGCCGCGAAGATCAACAAAGTATTCAG	TTTCTTGAGTTCGGTGATCCCAATG
AAA.8	GCGGCCGCGAAAGTATTCAGTACCCCCATAC	GCTTTCCAGTTTCTTGAGTTC
AAA.9	GCGGCCGCGAGTACCCCCATACCCTTTTCG	GTTGATCTTGCTTTCCAGTTTC
AAA.10	GCGGCCGCGATACCCTTTTCGTATAGCTAAC	GAATACTTTGTTGATCTTGC
AAA.11	GCGGCCGCGTCGTATAGCTAACTCGAGGGATC	GGGGGTACTGAATACTTTGTTG
AAA.12	GCGGCCGCGTAACTCGAGGGATCCGAATTC	AAAGGGTATGGGGGTACTG

### ELISA

The reactivity of rAbs and antibodies in LCL supernatant was assessed against each antigen by ELISA. To detect reactivity against TeNT, 1 µg/mL of Lc, Hn, Hc, TeNT (gifted from Dr. Masaaki Iwaki at the National Institute of Infectious Diseases), TeNT mix (a mixture of Lc, Hn, and Hc proteins), and tetanus toxoid were used for capture, and ELISA was performed as previously described^22^. To measure the concentration of human immunoglobulin in LCL supernatant, 10 µg/mL anti-human IgG (SouthernBiotech, Birmingham, AL, USA) was used for capture and ELISA was performed as previously described^23^. To determine the binding region of 8D8, each Hn mutant protein lysate was used for capture, along with 1 µg/mL of 8D8.

### Hc-GT1b binding assay

The inhibitory effect of antibodies on Hc-ganglioside GT1b binding was analyzed in an Hc-GT1b binding assay. Fifty microliters of 1 µg/mL ganglioside GT1b (Adipogen Life Sciences, San Diego, CA, USA) were dispensed into 96-well microtiter plates (Thermo Fisher Scientific) and the solvent methanol was evaporated for 8 h at room temperature. After washing three times with PBS-0.1% Tween 20 (PBS-T), the plates were blocked with 2% bovine serum albumin and PBS for 1 h at room temperature. Fifty microliters of 5 µg/mL Hc were mixed with serially diluted 50 µL of LCL supernatant or medium and incubated for 1 h at 37 °C. The plate was washed once with PBS-T, and each preincubated Hc/antibody mixture was added to the wells and incubated for 1 h at room temperature. The plates were then washed three times and incubated with mouse anti-Hc (produced in our lab) for 2 h at room temperature. Alkaline phosphatase (AP)-conjugated goat anti-mouse IgG (SouthernBiotech) was used to detect Hc binding to GT1b. Two-way analysis of variance (ANOVA) for statistical analysis were performed using Prism software (GraphPad Software, San Diego, CA, USA). A p value less than 0.05 was considered statistically significant.

### In vivo TeNT neutralization assay

The TeNT-neutralizing ability of all antibodies was analyzed in vivo. To set the survival time with toxin administration to 48 h, we selected the TeNT dose at 1 LD_50_ (25 pg). TeNT was mixed with LCL supernatant containing 0.5 ng of antibody, or with 0.025, 0.125, 1.25, or 1250 ng of purified rAbs and the volume was brought to 400 µL with 2% gelatin, and PBS. After 1 h incubation at room temperature, each antibody was subcutaneously injected into the left femur of four ddY mice (5 weeks of age) and the mice were monitored for symptoms of paralysis. The probability curves for survival were calculated according to the Kaplan–Meier method and compared by the Log-rank test using Prism software (GraphPad Software). A p value less than 0.05 was considered statistically significant. The protocols of animal experiments were approved by the Animal Experimentation Committee of the Research Institute for Microbial Diseases, Osaka University (approval number H30-16–0). The authors complied with the ARRIVE guidelines.

### Construct of Ig expression vector

To construct the expression vector for monoclonal antibodies, total RNA was first isolated from LCLs using a miRNeasy Micro Kit (Qiagen, Hilden, Germany) according to the manufacturer’s protocol. Then nested RT-PCR was performed using a SMART cDNA Library Construction Kit according to the manufacturer’s protocol (Takara). The following primers were used for the nested RT-PCR: IgH (IgG1), 1st forward primer (SMART 1st): 5’-AAGCAGTGGTATCAACGCAGAGT-3’ and 1st reverse primer: 5’-CGGGGAGCGGGGGCTTGCCGGCCGTCGCAC-3’; 2nd forward primer: 5’-GGGGCGGCCGCAGAGTGGCCATTACGGCCGGG-3’ (SMART 2nd) and 2nd reverse primer: 5’-GGGGAATTCTCATTTACCCGGAGACAGGG-3’: IgL (Igk), 1st forward primer: SMART 1st and 1st reverse primer: 5’- ACTGAGGAGCAGGTGGGGGCACTTCTCCCT-3’; 2nd forward primer: SMART 2nd and 2nd reverse primer: 5’-GGGGAATTCCTAACACTCTCCCCTGTTG-3’. The PCR-amplified fragment was digested with Not I and EcoR I and cloned into a human EF1α promoter-containing pQEFIP vector or pQEFIN vetctor that was derived from pQCXIP or pQCXIN vector (Takara), resplectively. The cloned immunoglobulin genes of 8A7, 17F7, 8D8, and 16E8 were confirmed to be composed of a combination of IgG1 and Igκ by DNA sequencing.

### Expression and purification of recombinant antibodies

Transient expression of recombinant Ig was performed using the Expi293 Expression system (Thermo Fisher Scientific). Expi293F cells were cotransfected with a mixture of IgH and IgL expression vectors according to the manufacturer’s protocol. Following culture of the transfected cells for 7 days, the culture supernatants were loaded onto a HiTrap Protein G HP Columns (Cytiva). Sample preparation on the column continued according to manufacturer-suggested protocols, resulting in the solubilized preparation of recombinant monoclonal antibodies. The purity of the purified recombinant monoclonal antibodies was confirmed by SDS-PAGE.

### Analysis of immunoglobulin genes

Analysis of the immunoglobulin gene usage, CDR3 amino acid sequences, and the number of amino acid mutations was performed using IMGT/V-QUEST at the web site of the International Immunogenetics Information System (http://www.imgt.org/IMGT_vquest/vquest).

### Antibody structure prediction and antibody-antigen docking

The 3-D structure of the variable domain of an 8D8 antibody clone was predicted using locally installed Rosetta software (version 2020.37) (http://www.rosettacommons.org/)^24^. Briefly, amino acid sequences of light (V_L_) and heavy (V_H_) chain variable domains of the antibody clones were used for a BLASTp search against the Protein Data Bank (PDB) database, in order to generate CDR-grafted antibody models as templates. Two-hundred runs of antibody structure modeling were performed using RosettaAntibody software with the generated templates, and the most suitable antibody template was selected according to the modeling results. For antibody-antigen docking simulation, the 3-D structure of full-length TeNT (PDB ID: 5N0B) composed of Lc, Hn, and Hc domains was downloaded from the PDB website (https://www.rcsb.org/)^12^. Two-hundred runs of antibody-antigen docking simulation were performed using SnugDock software with the obtained antibody and TeNT structures, and the most suitable docking model was selected according to the results of the docking simulation and the ELISA-based epitope mapping. The selected docking model was visualized and analyzed using UCSF Chimera with default settings^25^.

## Data Availability

The authors confirm that the data supporting the findings of this study are available within the article.
